# Transgenerational Plasticity in Flower Color Induced by Caterpillars

**DOI:** 10.3389/fpls.2021.617815

**Published:** 2021-03-15

**Authors:** Mar Sobral, Isabelle P. Neylan, Eduardo Narbona, Rodolfo Dirzo

**Affiliations:** ^1^Departamento de Biología Funcional, Universidade de Santiago de Compostela, USC, Santiago de Compostela, Spain; ^2^Department of Biology, Stanford University, Stanford, CA, United States; ^3^Department of Evolution and Ecology, Center for Population Biology, University of California, Davis, Davis, CA, United States; ^4^Departamento de Biología Molecular e Ingeniería Bioquímica, Universidad Pablo de Olavide, Seville, Spain; ^5^Woods Institute for the Environment, Stanford University, Stanford, CA, United States

**Keywords:** flower color, methylation, transgenerational plasticity, herbivory, anthocyanins, chromatin modifications, Brassicaceae

## Abstract

Variation in flower color due to transgenerational plasticity could stem directly from abiotic or biotic environmental conditions. Finding a link between biotic ecological interactions across generations and plasticity in flower color would indicate that transgenerational effects of ecological interactions, such as herbivory, might be involved in flower color evolution. We conducted controlled experiments across four generations of wild radish (*Raphanus sativus*, Brassicaceae) plants to explore whether flower color is influenced by herbivory, and to determine whether flower color is associated with transgenerational chromatin modifications. We found transgenerational effects of herbivory on flower color, partly related to chromatin modifications. Given the presence of herbivory in plant populations worldwide, our results are of broad significance and contribute to our understanding of flower color evolution.

## Introduction

Flower color is a key trait in angiosperm evolution. It is one of the most conspicuous traits under natural selection because of its potential role in attracting pollinators, and the subsequent influence on reproductive fitness (Spaethe et al., 2001; Wester and Lunau, 2017). Flower color is commonly related to quality or quantity of floral rewards, such as nectar or pollen (Fenster et al., 2004; Rausher, 2008). This relationship opens up the possibility of evolutionary diversification driven by pollinators acting on flower color variation within a species (Gegear and Burns, 2007; Losada et al., 2015; Sobral et al., 2015; Narbona et al., 2018).

Color in flowers is mainly due to the presence of pigments (Van Der Kooi et al., 2019). Among plant pigments, flavonoids—specifically anthocyanins—are the most common and diverse type (Tanaka et al., 2008). However, besides pollinator attraction, anthocyanins, and other groups of flavonoids that share a common biosynthetic pathway, may act as deterrents or defensive compounds against floral antagonists (Lev-Yadun and Gould, 2008; Zhang et al., 2013). Thus, herbivores, seed predators, and pathogens could also play an important role as non-pollinator agents of selection in flower color (Strauss and Whittall, 2006; Veiga et al., 2015). In addition, abiotic factors may also shape flower color variation (Dalrymple et al., 2020).

Flower color polymorphism within a species occurs when more than one flower color morph is present among individuals in the same or different populations (Warren and Mackenzie, 2001; Narbona et al., 2018). Causes of flower color polymorphism include different selection pressures imposed by mutualistic pollinators, antagonistic herbivores, or pathogens (Veiga et al., 2015; Narbona et al., 2018). This process is supposed to require the establishment and maintenance of a flower color mutant genotype in the population (Sobel et al., 2010; Del Valle et al., 2019) with the flower color variation based on the downregulation of structural or regulatory genes of the biosynthetic pathway of pigments accumulated in flowers (Sobel and Streisfeld, 2013; Roberts and Roalson, 2017). Thus, flower color polymorphism is expected to involve changes in nucleotide sequence that are transmitted to subsequent generations. Recently, it has been found that a radical change in flower morphology, including petal color, in *Moricandia arvensis* is due to within-individual plasticity produced by seasonal changes in climatic conditions (Gómez et al., 2020). A transcriptomic analysis found a coordinated response of more than 600 genes that was differentially expressed between two types of flowers suggesting a genetic basis for this plastic response (Gómez et al., 2020; see also Laitinen and Nikoloski, 2019).

As flower color is a trait highly affected by evolutionary constraints due to the influence of pollinator and non-pollinator agents of selection (Schiestl and Johnson, 2013; Sletvold et al., 2016), plasticity in flower color is expected to be low in comparison with other floral and vegetative traits (Del Valle et al., 2018, 2019; but see Gómez et al., 2020). However, flower color may show some degree of plasticity in response to abiotic as well as to biotic stressors, such as herbivores or pathogens (Wang et al., 2017; Rusman et al., 2019b; Koski and Galloway, 2020). Beyond this within-generational plasticity, it is not yet known whether there are possible transgenerational plastic effects of herbivory on flower color. In other words, we do not know if flower color plasticity is influenced by herbivory events across generations. Exploring whether the ecological experiences of previous generations, such as herbivory, influence flower color may contribute to our understanding of flower color evolution.

Transgenerational phenotypic plasticity challenges our current knowledge of evolutionary processes. Environmental factors can modify phenotypes directly via epigenetic modifications (Jablonka and Raz, 2009) that are transmissible across generations and can be adaptive (Jablonka and Raz, 2009; Herman and Sultan, 2016). Such phenotypic modifications can be manifested in a variety of ecologically relevant traits (Bossdorf et al., 2010), including the induction of defenses against herbivores (Herrera and Bazaga, 2013), and with subsequent effects on plant fitness (Herrera et al., 2014), at least partially independent of genetic variation (Verhoeven et al., 2010). Natural selection could act on this variation through its effect on ecological interactions that in turn affect fitness (Bossdorf et al., 2008; Alonso et al., 2019). In this way, transgenerational plasticity can influence the course of evolution (Bonduriansky and Day, 2009). Despite recent discoveries about the ecological and evolutionary implications of transgenerational effects (Verhoeven et al., 2016; Colicchio and Herman, 2020), transgenerational consequences of herbivory on flower color have not yet, to our knowledge, been explored.

**FIGURE 1:**
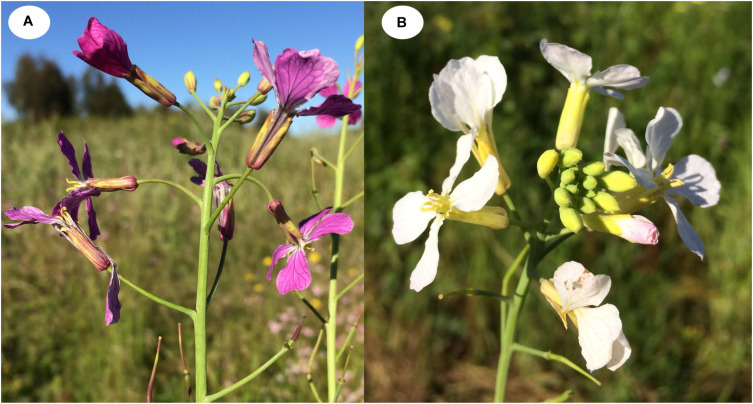
*Raphanus sativus* plants showing flower color polymorphism. **(A)** purple-flowered plants with anthocyanins present in petals. **(B)** White-flowered plants lacking anthocyanin in petals.

We conducted controlled experiments with the wild radish (*Raphanus sativus* L.) across four generations. This annual species shows a petal color polymorphism (Figure 1, white, pink, yellow, and bronze) due to the independent absence and presence of anthocyanins and carotenoids (Strauss et al., 2004; Strauss and Whittall, 2006). Here, we explore how the presence of petal anthocyanins across generations depends on herbivory by caterpillars of a specialized herbivore and whether such phenotypic changes are associated with chromatin modifications (i.e., genome-wide methylation).

## Materials and Methods

### Study Organisms

In California, wild radish, *Raphanus sativus* L. (Brassicaceae) is a naturalized, self-incompatible, herbaceous annual weed that grows along coastal sites, roadsides, and agricultural fields (Campbell and Snow, 2009). Wild radish has a flower color polymorphism that is driven by the presence or absence of anthocyanins (A) and carotenoids (C), with individuals showing either bronze (A+, C+), purple (A+, C−), yellow (A−, C+), or white (A−, C−) flowers (Irwin and Strauss, 2005). The inheritance of the pigments is Mendelian with two independently assorting loci coding for presence of anthocyanin and carotenoids (Irwin and Strauss, 2005).

Seeds germinate in October through November, and plants bloom in March for a period of 3–4 months. A wide variety of herbivores (aphids, snails, slugs, flea beetles, caterpillars, rabbits, and deer) feed on *R. sativus*. Prominent among these herbivores is the larval stage of the white cabbage butterfly, *Pieris rapae*, which was used for the herbivory treatments in this study. White cabbage butterfly caterpillars are specific herbivores to the Brassicaceae family.

### Experimental Design

We raised four generations of offspring from a single maternal family displaying white flowers collected from the wild in California. One maternal family (P) was raised in the greenhouse where they experienced no herbivory. After that, three generations (F_1_, F_2_, and F_3_) were raised. The plants used in this study are all descended from half siblings. To test our hypothesis, it was important to control for genetic variation so that any potential epigenetic or plastic mechanisms found could be interpreted and, at least partially, treated independently from genetic variation.

For all generations, we subjected plants to one of two treatments: herbivory or control (non-herbivory). We used a fully crossed, factorial experimental design, where the first factor was the exposure to herbivory in the F_1_ generation, the second was exposure in the F_2_ generation, and a third was exposure to herbivory in the F_3_ generation. In this way our F_3_ generation consisted of 130 plants in eight groups (between 12 and 22 plants per group) 68 of them with mothers attacked by herbivores and 62 of them with mothers not exposed to herbivores. This design enabled us to study the effect of herbivory across generations on subsequent flower color.

Plants were grown in germination flats in a greenhouse at the Stanford University Greenhouse Facility. We used a soil mix of 5% fine white sand, 20% potting soil and 75% peat moss (Orchard Supply Hardware, San Jose, CA, United States). Two weeks after germination, seedlings were transferred to 0.8-L pots. Plants were watered once a day and maintained at a 26°C/21°C, under a 12 h/12 h cycle. At the beginning of the experiment, the plants had two leaves. The caterpillars used were all second or third instar. The experiment lasted 2 weeks, until larvae started pupation. The amount of leaf tissue eaten was about 60% of the total leaf area on average but ranged from 40 to 80%. The caterpillars heavily attacked the plants and the plants quickly grew new leaf tissue in response. No plants were killed by the effects of herbivory. The caterpillars were purchased and shipped from Carolina Biological Company (CBC) and were delivered in small plastic containers with five to six larvae per container, and an agar and wheat-based food medium provided *ad libitum*. Caterpillars arrived during their second or third instar and were used in the experiments immediately upon arrival. Two *Pieris rapae* caterpillars were placed on the leaves of each plant in the herbivory treatment and allowed to roam around the plant freely for 2 weeks.

The herbivory treatment lasted for about the first quarter of the plants’ life. Plants germinated after 4 or 5 days of sowing the seeds, they flowered around 45 days later, and ended life with mature seeds at 2 months. Pollination was performed by hand with makeup brushes. In each generation, plants from the same treatment were crossed. We collected pollen with the brush from all plants per group and we pollinated all plants within the same group. For the F_3_ generation, four groups with 24 to 44 plants per group depending on the F_1_ and F_2_ treatment combination, were used. The goal was to pollinate all flowers on each plant. Several seeds per plant were sown and when all plants had germinated seeds, one seedling from each mother was selected using seedlings with similar sizes for all plants. We did not observe any plants with bronze flowers, and yellow forms were rare; thus, we focused on the production of anthocyanins (and we did not study carotenoid plasticity). Petal color of individuals was visually recorded as purple or white (i.e., presence or absence of petal anthocyanins, respectively; Figure 1). Biochemical analysis of white petals of *R. sativus* performed with HPLC-DAD showed absence of anthocyanins (E. Narbona et al. unpublished results).

### Chromatin Analyses

We collected leaf material from F_2_ generation plants before and after treatment. Plants were harvested at approximately 1 week of age before the treatment, and again at 3 weeks of age after the treatment. Plants had two or three leaves in the first sampling and between 5 and 10 leaves in the second sampling. Only fresh leaves that had recently expanded were sampled. We cut out one 2.7 cm diameter disc with a cork borer from a leaf, and leaf material was kept in dry silica gel until DNA extraction. The epigenetic characterization of the individuals before and after the treatment was made by means of methylation sensitive amplification fragment length polymorphism. In this technique, genomic DNA is digested by methylation-sensitive enzymes providing an epigenetic fingerprint for the plants.

We were interested in detecting DNA methylation events experienced by individual genotypes (and not on methylation differences between genotypes). Thus, a simplified MSAP method was performed using only primer combinations with the methylation-sensitive *Hpa*II. *Hpa*II cleaves CCGG sequences when cytosines are not methylated. Cleaving may be impaired when at least one cytosine is hemi-methylated and this process is inactive if one (or both) of the cytosines are fully methylated (Herrera et al., 2012). Thus, within the same genotype, polymorphism of MSAP markers reflects variation in the methylation status of the CCGG sites. The change from presence to absence implies a methylation event in a locus, and the change from absence to presence implies a de-methylation event (see Herrera et al., 2012 for an application of this simplified MSAP method).

DNA was isolated using the hexadecyl-trimethyl-ammonium-bromide procedure. After that, MSAFLP fingerprinting was performed. We first performed a pilot study with 15 individuals (30 samples) and 6 primer combinations. Based on this approach, we analyzed 106 samples of 53 plants belonging to the F_2_ generation using 2 primer combinations. The selected combinations X-AC/M-AC and X-AC/M-ATC were used for fragment amplification. MS-AFLP analyses were carried out by Keygene Laboratories (Netherlands).

### Statistical Analyses

#### Transgenerational Effects of Herbivory on Flower Color

A generalized linear model (binomial error distribution, link logit) for the 130 plants from F_3_ was performed to relate the anthocyanin presence (with or without anthocyanin) to the herbivory treatment in F_1_, F_2_, and F_3_ generations. We additionally tested this model including two- and three-way interactions among generation treatments. An alternative model was tested in which the fixed factors included the number of herbivory events across generations and the order of the herbivory treatments across generations (nested in number of herbivory events). All models were compared by means of AICc criterion and the model which presented the best fit included exclusively the herbivory treatment in each generation.

#### Transgenerational Effects of Maternal Methylation on Flower Color

Only fragments >300 base pairs in size were considered to reduce the potential impact of size homoplasy (Vekemans et al., 2002). Methylation and de-methylation cannot occur at the same time. Therefore, we considered non-methylation events only when de-methylation did not occur on the marker. MSAP marker scores for samples were transformed by comparing with the corresponding values (i.e., same plant individual before the treatment). MSAP marker scores involving a change from 1 to 0 denoted a methylation event of the marker involved. A new sample (*N* = 53 plant individuals) by marker (*N* = 402) score matrix was obtained where each element denoted whether the sample involved had experienced a methylation event (score = 1) or not (score = 0) in the corresponding marker (or missing when a de-methylation event occurred or when loci were already methylated before the treatment and therefore a methylation event was not possible). We assessed the genome-wide methylation per individual as the percentage of methylation events occurring per individual during treatment.

To understand the potential mechanism of transgenerational plasticity in flower color, we analyzed how methylation in F_2_ plants affected flower color in the F_3_ generation. A generalized linear model (binomial error distribution, link logit) for the response variable anthocyanin presence or absence (1/0) in the F_3_ generation was performed to relate this pigment presence to the global methylation of their mothers during treatments (fixed covariate included in the model). We tested alternative models in which herbivory treatments across generations were also included either independently or as the number of inductions in the ancestry line and we selected the model in which only the covariate methylation was included based on the AICc criterion.

Statistical analyses were performed in RStudio for R version 4.0.2 (R Core Team, 2013). Models were fitted using the function glm from the lme4 package (Bates et al., 2015). Statistical significance of fixed effects was determined by analysis of deviance, Type II Wald chi-square tests using the ANOVA function from the car package and the function AICc from the AICcmodavg package.

## Results

### Transgenerational Effects of Herbivory on Flower Color

**FIGURE 2:**
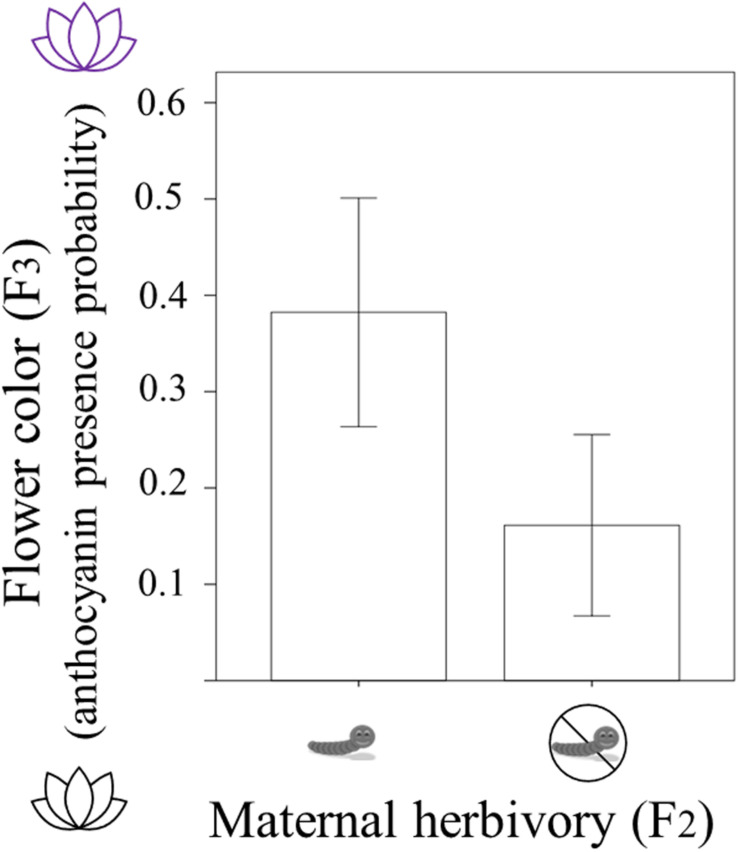
Flower color of *Raphanus sativus* F_3_ plants (with white flowers having no anthocyanins and purple flowers presenting anthocyanin; y-axis) depending on whether their mothers experienced herbivory by caterpillars (maternal herbivory in F_2_; x-axis). Difference is statistically significant (*P* < 0.01) and bars correspond to two standard errors.

In F_3_ plants, 94 individuals had white flowers (without anthocyanins) and 36 individuals had pink/purple flowers (with anthocyanins). The presence/absence of anthocyanins in these 130 (F_3_) individuals was related to the maternal herbivory treatment (Table 1). Plants were more likely to present purple flowers (with anthocyanins) when their mothers had suffered herbivory. The probability of producing petal anthocyanins was around 16% in plants coming from mothers who experienced no herbivory whereas it was around 38% in plants whose mothers were attacked by herbivores (Table 1 and Figure 2). Current and grandmaternal herbivory were not related to flower color and the interactions between generation treatments were not retained in the model (and non-significant).

**TABLE 1:** Results of the generalized linear model analyzing flower color (probability of F_3_ individuals containing anthocyanin in their petals) as a function of the herbivory in F_1_, F_2_, and F_3_ generations.

Response variable		Estimate	s.e.	*z*-value	*P*-value
	Current herbivory	–0.329	0.407	–0.809	0.418
Flower color in F3	**Maternal herbivory**	–1.139	0.4316	–2.641	**0.008**
(anthocyanin presence)	Grandmaternal herbivory	0.235	0.421	0.558	0.577

**Significant P-values are highlighted in bold.**

### Transgenerational Effects of Maternal Methylation on Flower Color

**FIGURE 3:**
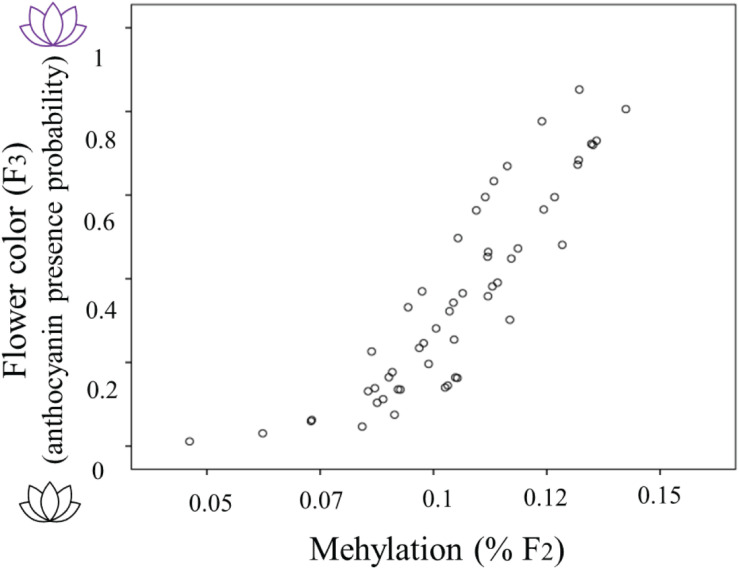
The relationship between flower color of *Raphanus sativus* F_3_ plants (purple flowers producing anthocyanins and white flowers lacking anthocyanins) with the genome wide percent methylation in their F_2_ mothers. Flower color is estimated as the predicted probability of anthocyanin presence from the saturated model including herbivory treatments and methylation (see “Statistical Analyses” section for details).

The occurrence of methylation during the 2-week herbivory/non-herbivory treatment ranged from 5 to 14% of genome wide loci. The presence of anthocyanin in plants from the F_3_ generation was related to the percent genome-wide methylation of their mothers (Estimate 61.069, s.e. 21.127, Z = 2.891, *P* = 0.004). Global genome-wide cytosine methylation of F_2_ plants was positively related to the probability anthocyanins presence in their offspring (Figure 3). The model which included herbivory across generations and methylation in F_2_ performed worst in terms of AICc score, thus it is not presented. When including simultaneously both F_2_ methylation and maternal herbivory, the latter was not significant.

## Discussion

Within-generational plasticity in flower color as well as the role of epigenetic inheritance in flower color variation have been recently reported (Gómez et al., 2020; Han et al., 2020). However, little work has focused on the possible transgenerational nature of plasticity in flower color and its potential eco-evolutionary causes. Here, we show that flower color may be linked to the herbivory, and methylation, in the previous generation. Flower color is not only a trait affected by the biotic and abiotic environment through natural selection and plasticity (Rusman et al., 2019a; Dalrymple et al., 2020), but our results indicate that flower color variation, at least in some cases, may be related to herbivory induced transgenerational plasticity.

Our results also indicate that this transgenerational effect of herbivory on flower color in *R. sativus* may be related to chromatin modifications. Although there are some reports of epigenetic inheritance involved in flower color modification in ornamental plants (Deng et al., 2015; Morita and Hoshino, 2018), this phenomenon has remained virtually unknown in wild plants, with some exceptional, related instances. For example, it has been recently proposed that epigenetic regulation of anthocyanin production may take place under stress-induced conditions in leaves of *Arabidopsis* and poplar (Fan et al., 2018; Cai et al., 2019). The regulation of the expression of a set of anthocyanin biosynthetic genes is mediated thorough the SWR1 ATP-dependent chromatin remodeling complex (Cai et al., 2019). The anthocyanin biosynthetic pathway in floral and foliar tissues shares the same structural genes, and differs only in the identity of transcriptional regulators (Hichri et al., 2011; Albert et al., 2014), opening up the possibility that a similar epigenetic process to that found in *Arabidopsis* and poplar could be acting in *R. sativus*. In fact, changes in methylation levels in an anthocyanin biosynthesis transcription factor have been recently found in petals of *Malus halliana* during flower color fading (Han et al., 2020).

Herbivory had previously been shown to relate to flower coloration, but this result was interpreted as defense traits pleiotropically linked to flower color morph because of the relationship between glucosinolates and the anthocyanin biosynthetic pathway (Strauss et al., 2004; Strauss and Whittall, 2006). Here we find that this relationship may be due to anthocyanins and flower color being plastic themselves, and responding to the same stimulus as plant defenses, expanding our previous understanding of this process. A possible interpretation of our results is that glucosinolates are upregulated in a transgenerational way, and anthocyanins are therefore indirectly induced by herbivory. In addition, anthocyanins share biosynthetic pathway with other flavonoids with well-known functions in plant defense, such as isoflavonoids, flavones, flavonols, and proanthocyanidins (Gould and Lister, 2006). This opens up the possibility that an upregulation of plant defense flavonoids in flowers may pleiotropically increase anthocyanin production due to their common biosynthetic origin (Johnson et al., 2015).

The ecological significance of the increase in probability to produce purple flowers lies in the complex pattern of mutualistic and antagonistic interactions this species shows. For example, pollinators, primarily honeybees, prefer to visit flowers of *R. sativus* with an absence of anthocyanin (white and yellow) (Stanton, 1987; Irwin and Strauss, 2005). Additionally, it has been found that herbivore damage in *R. sativus* can induce resistance to florivores in petals. Florivores prefer petals from plants which have not been previously attacked, but this difference is only noticeable in anthocyanin free plants (McCall et al., 2018). We have previously reported (Neylan et al., 2018) that the number of herbivory events across generations affected plant palatability to generalist slugs, but not to specialized caterpillars. Flower color variation seems therefore to be related to transgenerational effects of herbivory and plant and flower palatability (McCall et al., 2018; Neylan et al., 2018). The ecological implications of these multi-generational, multi-faceted effects may affect not only the response-inducing antagonists (herbivores) but mutualists as well, with complex and far-reaching consequences at both the population and community level (Jacobsen and Raguso, 2018; Rusman et al., 2019b; Kessler and Chautá, 2020).

The consequences of transgenerational plasticity through shared pathways and potential pleiotropies with traits that affect reproductive capabilities in offspring, remain understudied but potentially significant (Lehto and Tinghitella, 2020). Our work suggests transgenerational effects of herbivory, related to epigenetic modifications, on flower color variation within a species, improving our understanding of flower color evolutionary processes.

## Data Availability Statement

The raw data supporting the conclusions of this article will be made available by the authors, without undue reservation, to any qualified researcher.

## Author Contributions

MS and RD designed the study. MS and IN analyzed the data and performed the experimental work. MS and EN wrote the manuscript. All authors contributed to the revisions.

## Conflict of Interest

The authors declare that the research was conducted in the absence of any commercial or financial relationships that could be construed as a potential conflict of interest.
